# EFFICACY OF WATER INFUSION VERSUS AIR/CO_2_ INSUFFLATION FOR COLONOSCOPY: A SYSTEMATIC REVIEW AND META-ANALYSIS

**DOI:** 10.1590/S0004-2803.24612025-113

**Published:** 2026-05-25

**Authors:** Fernando RODRIGUEZ-GUZMAN, Anna Carolina Orsini ARMAN, Marcelo Morganti Ferreira DIAS, Diego PATERNOSTRO, Bruna Haueisen FIGUEIREDO-ZWETKOFF, Otávio MICELLI-NETO, José Celso ARDENGH

**Affiliations:** 1 Departamento de Diagnóstico por Imagem da Universidade Federal de São Paulo (UNIFESP), São Paulo, SP, Brasil.; 2 Serviço de Endoscopia Gastrointestinal, Hospital Moriah, São Paulo, SP, Brasil.; 3 Pontifícia Universidade Católica de Campinas, Faculdade de Medicina, Campinas, SP, Brasil.; 4 Departamento de Cirurgia e Anatomia do Hospital das Clínicas da Faculdade de Medicina de Ribeirão Preto (Universidade de São Paulo), Ribeirão Preto, SP, Brasil.

**Keywords:** Colorectal neoplasms, early detection of cancer, colonoscopy, insufflation, water, Neoplasias Colorretais, detecção precoce do câncer, colonoscopia, insuflação, água

## Abstract

**Background:**

Colonoscopy is a widely used screening method for colorectal cancer, playing a crucial role in early detection and prevention by allowing visualization and removal of precancerous lesions. It also helps diagnose and manage neoplastic lesions and inflammatory bowel disease by providing direct visualization of the intestinal mucosa. However, traditional air or carbon dioxide (CO_2_) insufflation may limit complete visualization of the colon. Alternatively, water infusion causes local distension without elongating the colon (unlike air insufflation), while warm water reduces spasms, decreasing insertion time and patient discomfort.

**Objective:**

This systematic review and meta-analysis aimed to compare water infusion versus air/CO_2_ insufflation in terms of technical efficacy, evaluate the effect of water immersion on procedural difficulty, and determine the accuracy of water immersion colonoscopy in detecting colon adenomas.

**Methods:**

We searched MEDLINE, EMBASE, and Cochrane CENTRAL databases for randomized controlled trials published from inception to January 2023. Outcomes included adenoma detection rate, success rate of cecal intubation, cecal intubation time, total procedure time (from insertion to withdrawal), need for abdominal compression, and on-demand sedation rate. Thirty randomized controlled trials were included.

**Results:**

adenoma detection rate, success rate of cecal intubation, cecal intubation time, and total procedure time showed no significant difference between the two methods (*P*>0.05). However, water infusion significantly reduced the proportion of participants requiring on-demand sedation (risk ratio 0.61, 95%CI 0.48-0.77, *P*=0.02) and abdominal compression (risk ratio 0.65, 95%CI 0.51-0.83, *P*<0.01).

**Conclusion:**

Colonoscopy with water infusion helps the colonoscope reach the cecum more easily, decreasing the need for on-demand sedation and abdominal compression.

## HIGHLIGHTS

• To evaluate whether colonoscopy with water infusion improves technical indicators and patient comfort when compared to air/CO2 insufflation.

• Systematic review and meta-analysis of 30 randomized controlled trials (7,909 patients) comparing adenoma detection rate, cecal intubation rate, procedure time, abdominal compression and on-demand sedation.

• Water infusion did not increase adenoma detection rate or cecal intubation rate, but reduced on-demand sedation (RR 0.61) and abdominal compression (RR 0.65), improving comfort.

• Although it does not improve all technical indicators, water infusion brings patient-centered benefits and can be indicated in selected cases.

## RESUMO

**Contexto:**

A colonoscopia é um método amplamente utilizado para detecção de câncer colorretal, desempenhando um papel essencial na prevenção e detecção precoce por meio da visualização e remoção de lesões pré-malignas. Além disso, esse método auxilia no diagnóstico e tratamento de lesões neoplásicas e doenças inflamatórias intestinais, proporcionando visualização direta da mucosa intestinal. No entanto, o uso de ar ou dióxido de carbono (CO_2_) pode limitar a visualização completa do cólon. Uma alternativa seria o uso de infusão de água, que produz distensão local sem alongamento do cólon (ao contrário da insuflação de ar), uma vez que a água morna minimiza os espasmos, reduzindo o tempo de inserção e o desconforto do paciente.

**Objetivo:**

Esta revisão sistemática e metanálise busca comparar a infusão de água com a insuflação de ar/CO_2_ no que se refere à eficácia técnica, além de avaliar o impacto da imersão em água na dificuldade do procedimento e determinar a precisão da colonoscopia por imersão em água na detecção de adenomas colorretais.

**Métodos:**

A busca foi realizada nas bases de dados MEDLINE, EMBASE, PubMed e Cochrane CENTRAL a fim de localizar ensaios clínicos randomizados publicados desde seu início até janeiro de 2023. Os desfechos incluíram a taxa de detecção de adenoma, a taxa de intubação cecal bem-sucedida, o tempo de intubação cecal, o tempo total de procedimento (tempo desde a inserção até a retirada), a necessidade de compressão abdominal e a taxa de sedação sob demanda. Trinta ensaios clínicos randomizados foram incluídos.

**Resultados:**

A taxa de detecção de adenoma, a taxa de intubação cecal bem-sucedida, o tempo de intubação cecal e o tempo total de procedimento não mostraram diferença significativa entre os dois métodos (*P*>0,05). No entanto, a infusão de água reduziu significativamente a proporção de participantes que necessitaram de sedação sob demanda (razão de riscos 0,61, IC95% 0,48-0,77, *P*=0,02) e compressão abdominal (razão de riscos 0,65, IC95% 0,51-0,83, *P*<0,01).

**Conclusão:**

A infusão de água facilita a inserção do colonoscópio no ceco, reduzindo a necessidade de sedação sob demanda e de compressão abdominal.

## INTRODUCTION

Colorectal cancer (CRC) is one of the leading causes of cancer-related death worldwide, and colonoscopy remains a key tool in its prevention and early detection^1^ ^-^ ^3^. This procedure allows visualization and removal of precancerous lesions, such as adenomas, which are crucial for reducing CRC incidence. However, colonoscopy effectiveness depends on several factors, including the method used for colonic distension. Traditionally, air or carbon dioxide (CO_2_) insufflation has been used to expand the colon. Still, recent studies suggest that water infusion techniques-such as water immersion and water exchange-may offer benefits for patient comfort, procedural efficiency, and adenoma detection rates (ADR)^4^ ^-^ ^6^. This raises an important question: does water infusion provide significant advantages over air/CO_2_ insufflation in colonoscopy? Specifically, do these differences lead to better patient acceptance, higher polyp detection, and less need for sedation?

This topic choice arises from the growing interest in improving colonoscopy outcomes while enhancing the patient experience. Current research indicates that water infusion techniques cause local distension^7^ without elongating the colon (unlike air insufflation)^8^. In contrast, warm water reduces spasms^9^, leading to shorter insertion times and less patient discomfort, decreasing the need for sedation, and potentially increasing ADR by improving mucosal visualization^10^ ^-^ ^12^. However, despite these possible advantages, the use of water infusion techniques remains limited, and the evidence supporting them is inconsistent. The most recent comprehensive review on this subject was published in 2015^13^, and since then, new studies have emerged that require an updated review of the literature. This knowledge gap underscores the need for a systematic review and meta-analysis to compare the efficacy of water infusion versus air/CO_2_ insufflation on key performance indicators, such as cecal intubation time (CIT)^14^, ADR, and patient comfort^15^ ^,^ ^16^.

The significance of this topic lies in its potential to improve both colonoscopy quality and patient outcomes. A higher ADR is associated with a lower risk of interval CRC, and each 1% increase in ADR is associated with a 3% decrease in CRC incidence^17^ ^-^ ^19^. Additionally, minimizing patient discomfort and the need for sedation could make colonoscopy more accessible and acceptable to a broader population, potentially boosting screening adherence^20^ ^,^ ^21^. Moreover, improving procedural efficiency by reducing CIT and loop formation might also decrease the risk of complications^22^, such as intestinal perforation and splenic injury^23^ ^,^ ^24^.

Given these considerations, this systematic review and meta-analysis aimed to test the hypothesis that, compared to traditional air/CO_2_ insufflation, water infusion techniques^25^ ^-^ ^29^ improve patient comfort, increase adenoma detection rates, and reduce the need for sedation during colonoscopy. By focusing on these outcomes, this review intends to inform clinical practice and support the adoption of techniques that enhance procedural quality and patient care.

## METHODS

This systematic review and meta-analysis was conducted following the Preferred Reporting Items for Systematic Reviews and Meta-Analyses (PRISMA) 2020 statement^30^. A comprehensive literature search was performed to identify published and unpublished randomized controlled trials (RCTs) comparing water infusion with air/CO_2_ insufflation in patients undergoing screening colonoscopy for CRC, regardless of whether it was their first procedure. The study protocol was registered with the International Prospective Register of Systematic Reviews (PROSPERO), registration number CRD42023439257.

### Study definitions

### Screening colonoscopy

The studies included in this systematic review and meta-analysis involved both first-time and subsequent screening colonoscopies. However, many studies did not explicitly distinguish between these groups.

### Study selection

Randomized controlled trials with the following characteristics were included in this systematic review and meta-analysis:

### Patient characteristics

The “Patient characteristics” described here refer to the eligibility criteria and demographic profiles of participants enrolled in the studies pre-selected for this systematic review. These studies were identified through a systematic search of clinical trial registries, and the reported inclusion and exclusion criteria were extracted to establish a consistent reference framework. This characterization does not represent a single patient population but is instead an aggregated overview of participant features across the selected trials. These data were used to contextualize and support the interpretation of subsequent outcome analyses presented in the main results.

Selected studies included male and female patients aged 18 years or older who were referred for diagnostic outpatient colonoscopy. They also included patients diagnosed with functional constipation according to the Roma III standard^3^ and those undergoing colonoscopy for routine health check-ups^5^. In contrast, they excluded individuals with inflammatory bowel disease, coagulation abnormalities, or on antithrombotic drug therapy; patients on hemodialysis with a bleeding tendency, or those with severe hepatic or renal disorders; patients with suspected hemodynamic instability, severe cardiovascular or pulmonary disease; individuals unable to communicate effectively, with a history of abdominal surgery, chronic benzodiazepine use, or who refused sedation; patients who refused or could not provide informed consent; those with inadequate bowel preparation, colonic stenosis, or poor bowel preparation (Boston Bowel Preparation Score <5 points); and patients classified as American Society of Anesthesiologists (ASA) III due to comorbid conditions.

Factors influencing procedural difficulty, such as those listed in the exclusion criteria, were not consistently reported across studies. These factors are known to significantly impact CIT, insertion difficulty, and patient comfort^12^ ^,^ ^17^ ^,^ ^18^.

### Intervention characteristics

Water, instead of air, was used to distend the colon sufficiently during insertion to ensure clear visualization of the lumen. Inclusion criteria: use of water infusion throughout the entire procedure (both insertion and withdrawal phases). Exclusion criteria: use of air insufflation during insertion; studies where water was used only for the sigmoid colon but not throughout the procedure; studies where water was instilled initially but not maintained consistently.

### Comparator characteristics

Standard colonoscopy employs air or CO_2_ insufflation to expand the colonic lumen for optimal visualization and easier advancement of the colonoscope. Inclusion criteria: studies where air or CO_2_ insufflation was used throughout the procedure, defined as the conventional technique. Exclusion criteria: studies where water immersion or water exchange replaced continuous air or CO_2_ insufflation.

### Endoscopist experience 

Endoscopist experience was inconsistently reported across studies, which may be a limitation. One study^20^ looked at trainee endoscopists’ learning progress over a 15-year training program. At the same time, another^31^ did not explicitly specify how experience was measured but indicated that ADR could serve as an indirect measure of endoscopist skill.

### Search strategy

The search strategy combined MeSH terms and natural language: “Colonoscopy” [MeSH Terms] AND “Insufflation” [MeSH Terms] AND “CO_2_” [All fields] AND “Air” [MeSH Terms] AND “Colorectal neoplasm/diagnosis” [MeSH Terms] AND (“Water exchanged” [All Fields] OR “water-assisted” [All Fields] OR “Underwater” [All Fields] OR “water-immersion” [All Fields] OR “water-aided” [All Fields]). The following electronic databases were searched to identify potential studies: MEDLINE, PubMed, Cumulative Index to Nursing and Allied Health Literature (CINAHL), Elton Bryson Stephens Company (EBSCO), Elsevier (Scopus), Excerpta Medica Database (EMBASE), Latin American and Caribbean Health Sciences Literature (LILACS), and Cochrane Central Register of Controlled Trials (CENTRAL). The results of the database searches were exported to Rayyan for title and abstract screening by two independent reviewers, using our inclusion and exclusion criteria.

### Eligibility criteria

Two independent reviewers (FRG and JCA) screened titles and abstracts using the PICO framework (patients, interventions, comparators, and outcomes): P - Patients undergoing screening colonoscopy for CRC; I - Water infusion colonoscopy; C - Air/CO_2_ insufflation colonoscopy; O - ADR, success rate of cecal intubation, CIT, total procedure time, need for abdominal compression, and on-demand sedation rate.

Full texts of all potentially eligible studies were retrieved and independently assessed by the same two reviewers.

Data were collected independently and entered into Excel spreadsheets. Any disagreements between reviewers were resolved through consensus or additional review by a third reviewer. Duplicates, prospective nonrandomized studies, case series, case reports, and studies not related to CRC screening were excluded.

Therefore, this review included RCTs published in English from the start of the database until January 2023 that reported on the results of a comparative analysis of water versus air methods in screening colonoscopy for CRC.

### Data extracted and outcomes 

For studies meeting eligibility, data collected included author, year of publication, study setting, indication for colonoscopy (screening), sample size and characteristics, type of intervention used (water immersion, water exchange, underwater), and results (outcomes). Outcomes measured included ADR, cecal intubation success rate, CIT, total procedure time, need for abdominal compression, and on-demand sedation rate. Subgroup analyses were performed on total procedure time, ADR, need for abdominal compression, and on-demand sedation rate. Each study was rated based on risk of bias, randomization, allocation, blinding, missing data, prognostic factors, outcomes, and number needed to treat (NNT)^32^ ^,^ ^33^.

### Risk of bias assessment

Risk of bias was assessed using the Cochrane risk-of-bias tool for RCTs (RoB 2), which evaluates 5 domains: Domain 1 (randomization process), Domain 2 (deviation from intended interventions), Domain 3 (missing outcome data), Domain 4 (measurement of outcomes), and Domain 5 (selection of the reported result)^32^. Blinding was not feasible due to the nature of the intervention, leading to bias in domains 2 and 5 (see Figure, SUPPLEMENTAL DIGITAL CONTENT 1, which provides a graphical overview of the risk of bias for each study). 

**SUPPLEMENTAL DIGITAL CONTENT 1.**
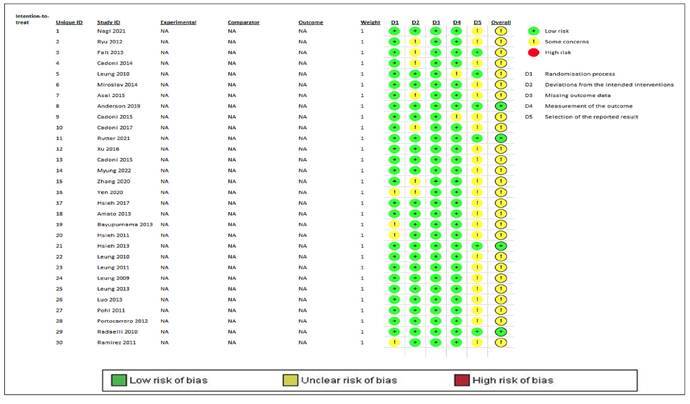
Graphical overview of risk of bias for each study included in the systematic review (Cochrane risk-of-bias tool for randomized controlled trials).

Note: the risk of bias presented above refers to the primary outcome of the study; no risk of bias was generated for secondary outcomes.

### Statistical analysis

Data from individual RCTs were combined for the final meta-analysis. All statistical analyses were conducted using R Studio, version 4.1. The pooled risk ratio (RR) and 95% confidence interval (CI) were calculated for dichotomous categorical variables to compare the groups. For continuous variables, the pooled mean difference between groups and its 95% CI were determined. Heterogeneity was assessed with the I2 statistic and categorized as follows: 0% to 40%, might not be important; 30% to 60%, moderate heterogeneity; 50% to 90%, substantial heterogeneity; and 75% to 100%, considerable heterogeneity. When significant heterogeneity was detected, the methods were examined to identify its source (33). Fixed-effect models were primarily used, with random-effect models applied when necessary. Forest plots illustrated the results and effect sizes. Since all data were available in the original outcome measures, no data imputation or transformation was conducted.

## RESULTS

### Study selection

The initial database search yielded 139 studies. After removing duplicates, screening titles and abstracts, and applying eligibility criteria, 30 RCTs were included in the final analysis^8^ ^,^ ^15^ ^,^ ^16^ ^,^ ^34^ ^-^ ^60^. The study selection process is shown in Figure 2.

**FIGURE 1:**
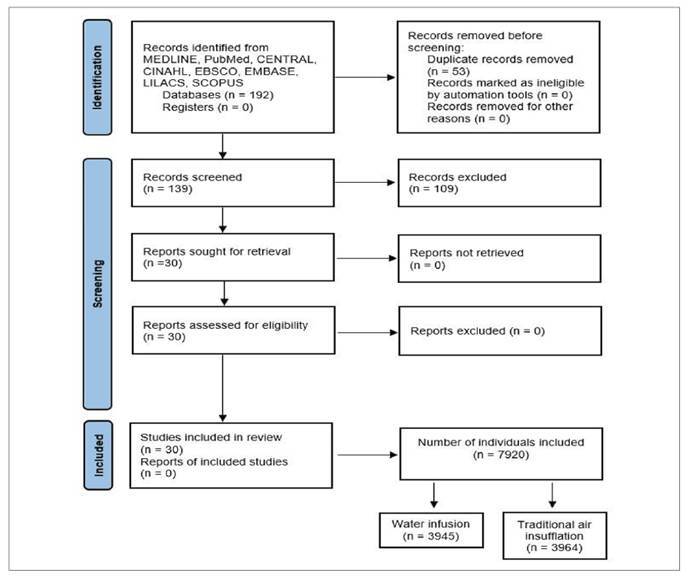
PRISMA flow diagram.

### Study characteristics

The 30 RCTs included in this review enrolled 7909 patients, with 3945 undergoing water infusion colonoscopy and 3964 undergoing air/CO_2_ insufflation colonoscopy. Most studies were conducted at centers in North America and Europe. Screening was the indication for colonoscopy in all cases. All patients were aged 18 years or older. Table 1 presents the characteristics of the studies included.

The results of the analysis of outcomes (ADR, on-demand sedation rate, success rate of cecal intubation, need for abdominal compression, CIT, and total procedure time) for the water infusion technique are summarized in Table 2.

**TABLE 1:** Synthesis of the characteristics of included studies.

Study	Country	Indication	Mean age (yrs)	Male participants (%)	Intervention
Nagl et al., 2021	Germany	Screening	67.2	52.9	Underwater
Ryu et al., 2012	South Korea	Screening	52.2	62.5	Instillation
Falt et al., 2013	Czech Republic	Screening	59.5	48.1	Immersion
Cadoni et al., 2014	Italy	Screening	59.0	60.3	Water-aided
Leung et al., 2010a	USA	Screening	61.8	100	Immersion
Miroslav et al., 2014	Slovenia	Screening	63.4	47.5	Immersion
Asai et al., 2015	Japan	Screening	58.7	67.4	Immersion
Anderson et al., 2019	USA	Screening	60.9	76.8	Underwater
Cadoni et al., 2015a	Italy	Screening	55.0	57.6	Water exchange
Cadoni et al., 2017	USA	Screening	54.6	54.9	Water exchange
Rutter et al., 2021	United Kingdom	Screening	59.5	50.0	Water assisted
Xu et al., 2016	China	Screening	58.0	47.6	Water infusion
Cadoni et al., 2015b	USA	Screening	56.3	58.5	Water exchange
Myung et al., 2022	South Korea	Screening	64.5	70.9	Underwater
Zhang et al., 2020	China	Screening	55.3	57.7	Underwater
Yen et al., 2020	USA	Screening	64.5	97.2	Underwater
Hsieh et al., 2017	USA	Screening	55.3	49.5	Water exchange
Amato et al., 2013	Italy	Screening	60.0	64.1	Water infusion
Bayupurnama et al., 2013	Indonesia	Screening	50.7	65.4	Water method
Hsieh et al., 2011	China	Screening	54.3	53.9	Water infusion
Hsieh et al., 2014	China	Screening	55.4	62.8	Immersion
Leung et al., 2010b	China	Screening	66.7	100	Water method
Leung et al., 2011	USA	Screening	59.5	99.0	Water exchanged
Leung et al., 2009	USA	Screening	59.5	91.1	Water exchanged
Leung et al., 2013	USA	Screening	60.5	97.0	Water infusion
Luo et al., 2013	China	Screening	56.2	30.9	Water method
Pohl et al., 2011	Germany	Screening	62.2	73.3	Water infusion
Portocarrero et al., 2012	USA	Screening	68.0	30.4	Water method
Radaelli et al., 2010	Italy	Screening	58.6	58.3	Water infusion
Ramirez et al., 2011	USA	Screening	59.6	96.4	Water method

**TABLE 2:** Water infusion colonoscopy - Analysis of outcomes.

Study	N	ADR	Success rate of cecal intubation	Need for abdominal compression	On-demand sedation	CIT (min)	Total procedure time (min)
Nagl et al., 2021	81	16	N/R	N/R	N/R	N/R	10.9
Ryu et al., 2012	51	N/R	47	N/R	N/R	6.92	N/R
Falt et al., 2013	101	41	90	37	5	6.9	16.4
Cadoni et al., 2014	338	136	326	N/R	39	11	21
Leung et al., 2010a	112	16	42	N/R	9	10.2	14.9
Miroslav et al., 2014	57	N/R	57	N/R	N/R	7.8	N/R
Asai et al., 2015	314	N/R	N/R	N/R	N/R	13.4	23.2
Anderson et al., 2019	60	27	N/R	N/R	N/R	6.5	17.3
Cadoni et al., 2015a	207	N/R	101	63	40	10.1	22
Cadoni et al., 2017	408	177	N/R	N/R	173	8	22
Rutter et al., 2021	561	N/R	N/R	N/R	N/R	N/R	8.83
Xu et al., 2016	97	13	N/R	21	N/R	6.9	14.9
Cadoni et al., 2015b	197	N/R	N/R	156	26	10	22
Myung et al., 2022	56	N/R	N/R	N/R	N/R	N/R	N/R
Zhang et al., 2020	66	N/R	N/R	N/R	N/R	N/R	N/R
Yen et al., 2020	128	116	128	N/R	N/R	N/R	N/R
Hsieh et al., 2017	217	88	N/R	157	88	7	19.3
Amato et al., 2013	113	44	N/R	N/R	15	9	17
Bayupurnama et al., 2013	53	N/R	N/R	N/R	N/R	11.9	18
Hsieh et al., 2011	51	15	N/R	19	N/R	5.6	15.3
Hsieh et al., 2014	90	41	89	59	N/R	6.5	N/R
Leung et al., 2010b	42	17	N/R	24	N/R	34	56
Leung et al., 2011	50	20	50	21	11	13.1	28.1
Leung et al., 2009	28	N/R	28	4	1.8	8.8	21
Leung et al., 2013	50	27	48	13	N/R	13	29
Luo et al., 2013	55	N/R	51	4	N/R	11.9	18.3
Pohl et al., 2011	58	19	48	30	5	8.1	19.2
Portocarrero et al., 2012	11	6	11	N/R	1.4	N/R	14
Radaelli et al., 2010	116	29	109	N/R	15	7	15
Ramirez et al., 2011	177	101	176	21	31	6.9	19.9

ADR: adenoma detection rate; CIT: cecal intubation time; N/R: not reported.

For the standard air/CO_2_ insufflation, the results are summarized in Table 3.

**TABLE 3:** Air/CO_2_ insufflation colonoscopy - Analysis of outcomes.

Study	N	ADR	Success rate of cecal intubation	Need for abdominal compression	On-demand sedation	CIT (min)	Total procedure time (min)
Nagl et al., 2021	76	19	N/R	N/R	N/R	N/R	8
Ryu et al., 2012	53	N/R	47	N/R	N/R	8.4	N/R
Falt et al., 2013	100	36	90	23	4	7	16
Cadoni et al., 2014	334	90	330	N/R	87	9	18
Leung et al., 2010a	114	21	45	N/R	24	15.2	20.9
Miroslav et al., 2014	65	N/R	65	N/R	N/R	8.6	N/R
Asai et al., 2015	325	N/R	N/R	N/R	N/R	13.2	22.9
Anderson et al., 2019	61	23	N/R	N/R	N/R	4.2	15.9
Cadoni et al., 2015a	208	N/R	102	78	44	9.5	12.85
Cadoni et al., 2017	408	165	N/R	N/R	195	8	20.5
Rutter et al., 2021	562	N/R	N/R	N/R	N/R	N/R	8.12
Xu et al., 2016	94	12	N/R	53	2	10.6	18.2
Cadoni et al., 2015b	193	N/R	N/R	164	58	12	23
Myung et al., 2022	54	N/R	N/R	N/R	N/R	N/R	N/R
Zhang et al., 2020	64	N/R	N/R	N/R	N/R	N/R	N/R
Yen et al., 2020	127	120	127	N/R	N/R	N/R	N/R
Hsieh et al., 2017	217	82	N/R	174	82	7.5	18.9
Amato et al., 2013	113	42	N/R	N/R	29	7	14
Bayupurnama et al., 2013	57	N/R	N/R	N/R	N/R	12.9	18.2
Hsieh et al., 2011	51	13	N/R	29	N/R	4.6	13.1
Hsieh et al., 2014	90	39	88	69	N/R	9.6	N/R
Leung et al., 2010b	40	11	N/R	34	N/R	37	56
Leung et al., 2011	50	18	50	37	23	11.0	24.4
Leung et al., 2009	28	N/R	28	11	2.5	11.0	21
Leung et al., 2013	50	24	44	27	N/R	12	28
Luo et al., 2013	55	N/R	42	21	N/R	11.5	17.7
Pohl et al., 2011	58	15	56	36	20	6.2	15.7
Portocarrero et al., 2012	12	2	12	N/R	3.8	N/R	16
Radaelli et al., 2010	114	46	109	N/R	25	5	14
Ramirez et al., 2011	191	88	191	54	52	5.3	18.9

ADR: adenoma detection rate; CIT: cecal intubation time; N/R: not reported.

### Adenoma detection rate

**FIGURE 2:**
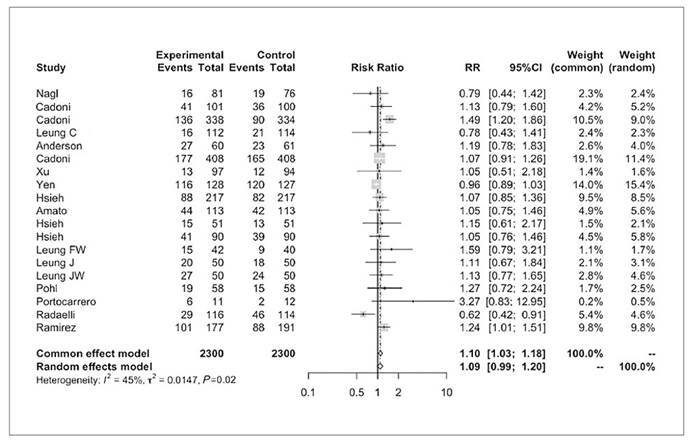
Forest plot of adenoma detection rate during colonoscopy with water infusion versus air/CO_2_ insufflation.

Nineteen studies assessed ADR, including a total of 2300 patients in the water infusion group and 2300 patients in the air/CO_2_ insufflation group (Figure 2)^8^ ^,^ ^16^ ^,^ ^34^ ^-^ ^50^. There were 947 adenoma detection events in the first group and 864 in the second group (RR 1.0, 95%CI 0.99 to 1.2, *P*>0.05). The NNT was 33.

### On-demand sedation 

**FIGURE 3:**
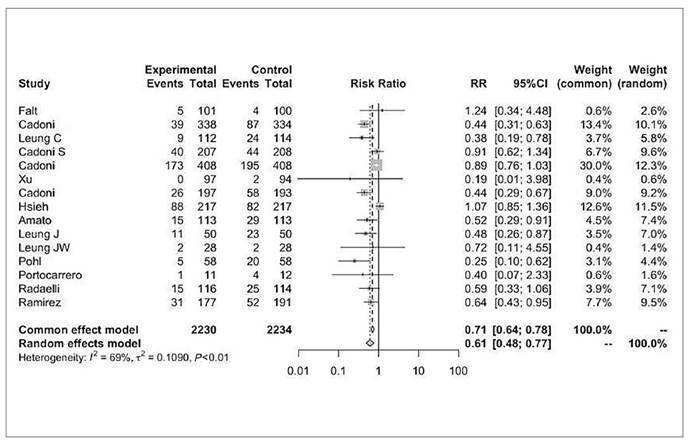
Forest plot of on-demand sedation rate during colonoscopy with water infusion versus air/CO_2_ insufflation.

Fifteen studies assessed on-demand sedation rates during colonoscopy, including a total of 2230 patients in the water infusion group and 2234 patients in the air/CO_2_ insufflation group (Figure 3)^8^ ^,^ ^16^ ^,^ ^35^ ^,^ ^36^ ^,^ ^38^ ^,^ ^39^ ^,^ ^41^ ^,^ ^42^ ^,^ ^46^ ^-^ ^49^ ^,^ ^51^ ^-^ ^53^. There were 460 events in the first group and 651 events in the second group (RR 0.61, 95%CI 0.48 to 0.77, *P*=0.02).

### Success rate of cecal intubation

**FIGURE 4:**
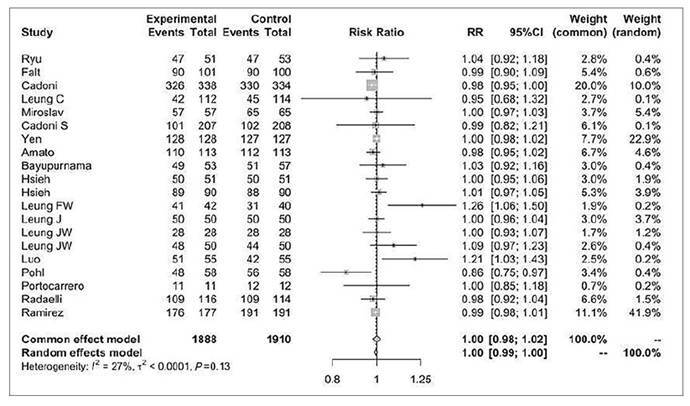
Forest plot of success rate of cecal intubation during colonoscopy with water infusion versus air/CO_2_ insufflation.

Twenty studies assessed the success rate of cecal intubation, involving a total of 1888 patients in the water infusion group and 1910 patients in the air/CO_2_ insufflation group (Figure 4) ^8^ ^,^ ^16^ ^,^ ^35^ ^,^ ^36^ ^,^ ^40^ ^,^ ^42^ ^-^ ^51^ ^,^ ^53^ ^-^ ^57^. There were 1651 events in the first group and 1670 events in the second group (RR 1.00, 95%CI 0.99 to 1.00, *P*>0.05).

### Need for abdominal compression

**FIGURE 5:**
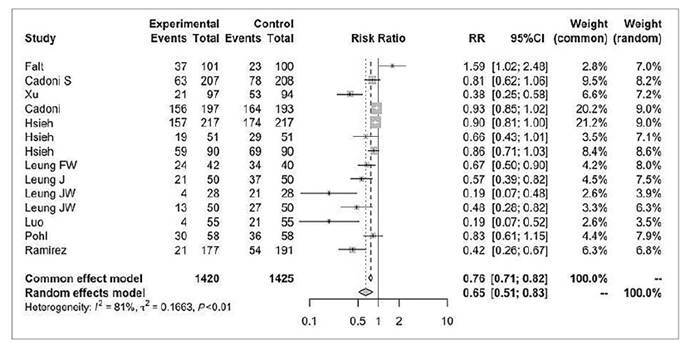
Forest plot of need for abdominal compression during colonoscopy with water infusion versus air/CO_2_ insufflation.

Fourteen studies assessed the need for abdominal compression during colonoscopy, involving a total of 1420 patients in the water infusion group and 1425 patients in the air/CO_2_ insufflation group (Figure 5)^8^ ^,^ ^16^ ^,^ ^39^ ^,^ ^41^ ^,^ ^43^ ^-^ ^46^ ^,^ ^49^ ^-^ ^53^ ^,^ ^57^. There were 629 events in the first group and 820 events in the second group (RR 0.65, 95%CI 0.51 to 0.83, *P*<0.01). The NNT was 20.

### Cecal intubation time

**FIGURE 6:**
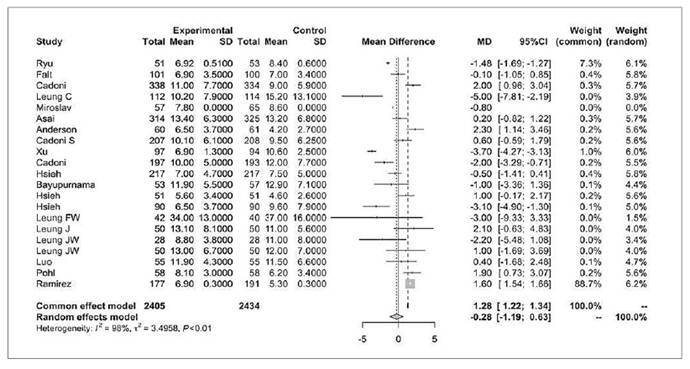
Forest plot of cecal intubation time (min) during colonoscopy with water infusion versus air/CO_2_ insufflation.

Twenty-one studies assessed CIT (in minutes), involving a total of 2405 patients in the water infusion group and 2434 patients in the air/CO_2_ insufflation group (Figure 6)^8^ ^,^ ^15^ ^,^ ^16^ ^,^ ^35^ ^-^ ^37^ ^,^ ^39^ ^,^ ^41^ ^,^ ^43^ ^-^ ^46^ ^,^ ^49^ ^-^ ^57^. A mean CIT was calculated for each group, and the mean difference was -0.28 (95%CI -1.19 to 0.63, *P*>0.05).

It showed no statistical significance.

### Total procedure time

**FIGURE 7:**
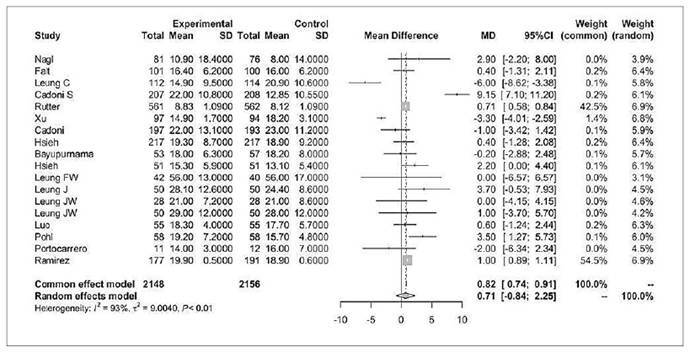
Forest plot of total procedure time (min) to complete colonoscopy with water infusion versus air/CO_2_ insufflation.

Eighteen studies assessed total procedure time (in minutes), defined as the duration from insertion to withdrawal of the colonoscope, involving 2148 patients in the water infusion group and 2156 patients in the air/CO_2_ insufflation group (Figure 7)^8^ ^,^ ^16^ ^,^ ^34^ ^,^ ^36^ ^,^ ^39^ ^,^ ^41^ ^,^ ^43^ ^,^ ^45^ ^-^ ^47^ ^,^ ^49^ ^,^ ^50^ ^-^ ^53^ ^,^ ^56^ ^-^ ^58^. A mean total procedure time was calculated for each group, and the mean difference was 0.71 (95%CI -0.84 to 2.25, *P*>0.05).

This outcome showed no significant difference.

Table 4 offers a summary of the comparative results between water infusion and air/CO_2_ insufflation across all variables of interest.

**TABLE 4:** Overall summary of the comparative results for the variables analyzed in colonoscopies with water infusion versus air/CO_2_ insufflation.

Variable	RCTs	Water infusion	Air/CO_2_ insufflation	RR (95%CI)	*P*
Adenoma detection rate	19	864	947	1.09 (0.99 to 1.20)	0.33
On-demand sedation	15	460	651	0.61 (0.48 to 0.77)	0.02
Success rate of cecal intubation	20	1651	1670	1.00 (0.99 to 1.00)	0.56
Need for abdominal compression	14	629	820	0.65 (0.51 to 0.83)	<0.01
Cecal intubation time (min)	21	Pooled mean	Pooled mean	-0.28 (-1.29 to 0.63)	0.56
Total procedure time (min)	18	Pooled mean	Pooled mean	0.71 (-0.84 to 2.25)	0.78

RCTs: randomized controlled trials. RR: risk ratio. CI: confidence interval.

## DISCUSSION

This systematic review and meta-analysis assessed the effectiveness of water infusion techniques in comparison to traditional air/CO_2_ insufflation during colonoscopy. The results indicate that although water infusion does not significantly reduce CITs^12^ ^,^ ^17^, it improves colon cleansing^13^, aids scope advancement^16^, and decreases patient discomfort^25^ ^,^ ^29^. Furthermore, water infusion is associated with lower rates of abdominal compression and on-demand sedation^8^ ^,^ ^22^, resulting in a better overall patient experience. Although water infusion has been shown to lessen pain and sedation needs, its effect on ADR remains uncertain. Some studies report improved ADR due to better mucosal visualization^38^ ^,^ ^61^, while others find no significant difference^41^. This variation suggests that water infusion techniques may offer specific benefits rather than a consistent advantage over air/CO_2_ insufflation.

The overall quality of evidence from the included RCTs is moderate, with notable methodological limitations. One of the main challenges encountered during the study selection process was the scarcity of trials that focused solely on air insufflation as the comparator. Despite its widespread historical use, few recent studies have isolated air as the only insufflation method for colonoscopy. As a result, many available studies either combined air with CO_2_ or compared water infusion with a mixed group, without clearly distinguishing between air and CO_2_. This lack of clarity leads to significant heterogeneity, especially since CO_2_ insufflation has been shown to reduce post-procedural discomfort compared to air^42^ ^,^ ^62^ substantially. Consequently, the inability to distinguish the effects of air from CO_2_ limits the interpretability of the findings and decreases the accuracy of comparisons involving water infusion techniques.

Another significant limitation is that endoscopist experience significantly affects procedural success, patient comfort, and complication rates^20^ ^,^ ^23^ ^,^ ^31^. The absence of experience stratification in many studies is a significant drawback. Differences in skill levels could influence comparisons between water infusion and air/CO_2_ insufflation techniques, potentially affecting the validity of the results^63^.

Additionally, blinding endoscopists was not feasible, which could have introduced performance bias in procedural assessments^30^ ^,^ ^32^. Differences in patient characteristics, such as prior abdominal surgery, diverticular disease, and irritable bowel syndrome, limit the generalizability of the findings^18^. Finally, the volume of water infused varied across studies, including 100-300 mL^28^ ^,^ ^54^ ^,^ ^64^, 241-521.4 mL^41^, and unrestricted volume of water^65^. This variation could have potentially influenced CIT^12^ ^,^ ^66^, ADR, and patient comfort^67^.

Results show that while water infusion techniques enhance patient comfort and lessen sedation requirements^15^ ^,^ ^16^, routine use for all colonoscopies may not be necessary. This method could be especially helpful for patients at high risk of sedation-related complications, such as those with cardiovascular or respiratory comorbidities^21^. Additionally, it may be beneficial during difficult colonoscopies, where looping and discomfort are more common^23^.

However, widespread adoption of water infusion may be limited by practical factors, including longer procedural times and the potential for higher costs from water leakage and additional equipment requirements^28^. The feasibility of incorporating water infusion into routine practice requires further assessment, especially in high-volume endoscopy centers where efficiency is essential^24^.

Given these limitations, future research should target several key gaps. First, subsequent trials should stratify outcomes by insufflation type, especially since CO_2_ insufflation has already shown benefits over air in reducing post-procedural discomfort^42^ ^,^ ^68^. This would offer a clearer view of the relative advantages and disadvantages of various insufflation methods. Second, there is a need to define optimal water volumes and infusion techniques, as standardization could improve reproducibility and clarify effects on ADR, CIT, and patient comfort^54^ ^,^ ^69^. Third, additional studies should examine whether specific subpopulations-such as older patients, individuals with prior surgeries, or those undergoing difficult colonoscopies-gain greater benefits from water infusion^23^ ^,^ ^56^. Identifying patient-specific advantages could lead to more personalized and effective colonoscopy practices. Lastly, it would be valuable to assess whether reduced pain and improved patient experience lead to greater adherence to CRC screening programs, with significant public health implications^14^. Exploring these long-term outcomes may help determine the broader impact of procedural improvements on screening compliance and overall public health.

## CONCLUSION

Water infusion during colonoscopy does not significantly increase cecal intubation rates compared to air insufflation. Still, it provides essential patient-centered benefits, such as reduced pain, reduced sedation requirements, and greater comfort, especially during procedures that do not require sedation. While it enhances colon cleansing and may help detect lesions, its effectiveness depends on patient factors and procedural differences. Standardizing water infusion techniques and conducting more research are necessary to improve its use. Despite some limitations, water infusion remains a promising addition to colonoscopy, potentially enhancing patient experience and screening compliance.

## Data Availability

Data in article: The research data are presented within the article itself (available in Tables 1-4, Figures 1-7 and Supplementary Material).
